# Evaluation of Nonmotor Symptoms in Diagnosis of Parkinsonism and Tremor

**DOI:** 10.1155/2016/9182946

**Published:** 2016-06-14

**Authors:** Andrew H. Evans, Chiun-Hian Chai

**Affiliations:** Department of Neurology, Royal Melbourne Hospital, 300 Grattan Street, Parkville, VIC 3050, Australia

## Abstract

*Background*. Nonmotor symptoms particularly olfactory dysfunction, RBD, depression, hallucinations, and constipation are currently not included in the typical clinical criteria for diagnosing Lewy body Parkinsonian disorders (LBPD). The aim of this study is to determine the diagnostic value of nonmotor symptoms in patients presenting with Parkinsonism and tremor.* Methods*. All new patients seen between January 2007 and May 2013 in the Movement Disorders Specialist Clinics of the Royal Melbourne Hospital (RMH), who were referred with a possible neurodegenerative syndrome or concerns of Parkinsonism and/or tremor, were included. Patients underwent routine evaluation with the four-minute “Sniffin Sticks” test, RBD, depression, and constipation.* Results*. 291 patients were included in the analysis.* Conclusion*. We found that lower olfaction scores based on “Sniffin Sticks” testing combined with reports of depression and constipation are independent predictors for the diagnosis of the spectrum of Lewy body Parkinsonian disorders (LBPD). Parkinson's disease (PD) cannot be reliably clinically differentiated from other causes of Parkinsonism that share symptomatology and structural abnormalities.

## 1. Introduction

There is still no universally reliable way to differentiate Parkinson's disease (PD) from other causes of Parkinsonism that share symptomatology and structural abnormalities. Several criteria to assist with PD diagnosis have been developed, including UK Parkinson's Disease Society (PDS) Brain Bank, National Institute of Neurological Disorders and Stroke (NINDS), and American Academy of Neurology (AAN) [1–3]. Until recently, these diagnostic criteria rely on clinical responsiveness to L-dopa and absence of features that suggest an alternative diagnosis.

It has been suggested that the diagnostic accuracy of neurologists with particular expertise in the field of movement disorders tends to exceed that claimed by clinical diagnostic criteria. Therefore, movement disorders specialists may be using a method of pattern recognition for diagnosis that goes beyond that inherent in any set of formal criteria [4]. It is likely that these clinicians use subtle, diagnostically valuable clinical clues to aid diagnosis, such as the pattern of nonmotor symptoms associated with early PD. These might include the presence of olfactory dysfunction, autonomic symptoms such as constipation, REM sleep behaviour disorder (RBD), and comorbid mood states.

Realization of the importance of nonmotor symptoms in Parkinson's disease has spurred international interest in the movement disorders community, which led to development of various screening and diagnostic tools to detect the presence of nonmotor symptoms and study their relationship with the development of PD. In fact, the recent EFNS/MDS-ES recommendations of the diagnosis of PD have included olfactory testing, RBD screening, and neuropsychiatric assessments at presentation as supportive features for PD but these criteria do not make specific mention of constipation [5].

The aim of this retrospective study is to identify patients referred to a movement disorders clinic and clinician's private rooms who underwent initial evaluation of olfaction (using Sniffin Sticks smell discrimination testing) in addition to routine initial evaluation for motor Parkinsonism and other nonmotor symptoms and compared to the final diagnosis after longitudinal clinical follow-up according to standard criteria. The relative values and the presence or absence of common nonmotor symptoms associated with PD and other Lewy body disorders were subsequently assessed.

## 2. Methods

### 2.1. Study Setting and Participants

All new patients seen between January 2007 and May 2013 in the Movement Disorders Clinics of the Royal Melbourne Hospital (RMH) referred with a possible neurodegenerative syndrome or signs of Parkinsonism and/or tremor were included. The referral source (primary care physician, general neurologist, or other specialists) was documented and basic patient demographics were recorded.

The routine initial clinical evaluation included the following:

1. The four-minute “Sniffin Sticks” test is a forced choice 12-item test, where a score <7 is defined as “anosmia,” >10 as “normosmia,” and score between 7 and 10 as “hyposmia” in the normative data [6]. Patients with self-reported loss of smell due to other causes were excluded.
2. It is assessment of the presence or absence of constipation according to self-report.
3. It is presence of self-reported depression or past treatment for depression.
4. It is presence of hallucinations according to the UPDRS part 1 [7].
5. It is presence of RBD, in which patients and carers were directly asked whether the patients acted out their dreams (e.g., running and flailing) or yelling in a manner similar to that recently described [8].

All patients had “Sniffin Sticks” evaluation of olfaction at initial evaluation. The initial referral symptom was obtained from the referring clinician's letter and divided into either Parkinsonism or primarily tremor. The movement disorders initial clinical diagnosis was defined after the first assessment and classified into five different groups, which includes Parkinson's disease (PD), Parkinson's disease dementia (PDD) and dementia of Lewy bodies (DLB), Parkinsonism and tremor disorders (PTDs), and unclassified parkinsonism (UP). These were compared to the final diagnosis made by the movement disorders specialists in clinic after longitudinal follow-up. Longitudinal follow-up was defined as all the visits after the initial visit within the study period. Where possible, patients at follow-up were classified according to standard clinical criteria [1, 9–11]. PDD was defined according to MDS guidelines [12].

This study was approved by the Melbourne Health Research and Ethics Committee.

### 2.2. Statistical Analysis

Statistical analyses were done with IBM SPSS Statistics version 19. Descriptive statistical analyses and chi square tests were performed on basic demographic characteristics, including gender and family history of Parkinsonism. Kruskal Willis nonparametric test was used to compare the age of symptom onset and Sniffin Sticks scores between groups, and PD was used as reference in* post hoc* analysis with Mann Whitney test. Bonferroni method was used to derive the level of significance of *p* < 0.0125.

At the time of initial assessment, Lewy Body Parkinsonian Disorder patients (LBPD) could develop into PD and PDD as well as DLB and therefore were grouped as LBPD group. The remaining patients were classified as non-LBPD group. The utility of Sniffin Sticks score and nonmotor symptoms were subsequently evaluated for their association with the final diagnosis. Binary logistic regression was used to derive the odds ratio (OR) for age of symptom onset, gender, Sniffin Sticks score and prevalence of RBD, depression, constipation, and hallucination between LBPD and the other non-LBPD group. Adjusted OR was then computed with binary logistic regression with hierarchical entry method.

## 3. Results

A total of 301 patients were identified during the study period, with 291 patients eligible for inclusion in the study. Ten patients were excluded due to lack of final diagnosis or concomitant significant sinusitis/head injury affecting olfactory function. The referral symptom was predominantly Parkinsonism in 220 patients and tremor in the remaining 71 patients. Main sources of referral were from general practitioner (53.3%) and other neurologists (21.6%). Other referral sources included physician (10.0%), surgeon (10.0%), and psychiatrist (5.2%). Mean duration of patients follow-up at movement disorder specialist clinic was 21.7 months (SD ± 20.4). Seventy-nine patients (27.1%) had at least one-year follow-up. LBPD patients had longer duration of follow-up with mean of 24.5 months (SD ± 20.9), compared to 18.1 months (19.3) in non-LBD group, *p* < 0.05.

### 3.1. Baseline Characteristics

All groups had proportionally more males than females except UP, but this group difference did not reach statistical significance (Table 1). Family history of Parkinsonism was in the range of 4.3% to 10.0% across groups, with 8.6% and 10.0% reported in PD and PDD/DLB groups, respectively. These results are similar to the figure of 10–20% reported in PD patients previously [13]. For the age of onset of motor symptoms,* post hoc* analysis with PD as reference group showed that PDD/DLB patients developed their first symptom at significantly older age, mean 69.7 years (SD ± 10.5).

### 3.2. Accuracy of Diagnosis by Referring Doctors and Movement Disorders Specialists after Long-Term Follow-Up

Compared to the diagnosis after follow-up, 50% of all LBD cases (76 out of 158 cases) were correctly diagnosed or suspected by the referring clinicians, as compared to 75% (119 out of 158) accuracy of initial diagnosis in movement disorder specialist clinic. Cases misdiagnosed as PD by referring doctors include MSA (3 cases), PSP (2 cases), ET (1 case), drug-induced Parkinsonism (1 case), vascular Parkinsonism (2 cases), UP (2 cases), and others (3 cases). In movement disorder specialist clinic, the main challenge at initial assessment was patients diagnosed with UP (82). Longitudinal follow-up of these patients identified PD and DLB (34), PTDs (total 21: MSA-3; PSP-4; ET-3; drug-induced Parkinsonism-6; vascular Parkinsonism-5), UP (17), and others (10) at final assessment.

### 3.3. Mean “Sniffin Sticks” Score

Mean “Sniffin Sticks” scores were the lowest in patients with PDD and DLB, 4.2 (SD ± 2.5), *p* < 0.05 (Table 2). This was followed by patients with PD, with mean “Sniffin Sticks” scores of 6.4 (SD ± 3.0). Mean “Sniffin Sticks” scores for PTDs, UP, and “other” groups were above 8. The results clearly showed that patients with LBPD (PD/PDD/DLB) performed poorly in “Sniffin Sticks” test and below the cutoff point of <7 for anosmia quoted in the normative dataset.

### 3.4. Prevalence of RBD

Fifty-nine patients were found to have symptoms of RBD. Prevalence of RBD was higher in LBPD group, 43 of 158 (or 27.2%, *p* < 0.05), even with MSA patients included in the non-LBPD group. Within the MSA patient group, 4 out of 9 patients (or 44.4%) were diagnosed to have RBD.

### 3.5. Unadjusted Odd Ratio in Association with LBPD Group

Subjects were classified into LBPD group and non-LBPD group for analysis in Table 3. Mean age of symptom onset was higher in LBPD patients, 63.5 years (SD ± 12.6), compared to non-LBPD patients, 59.2 years (SD ± 16.1) (OR 1.02; 1.00–1.04; *p* = 0.014). The proportion of male patients was higher in LBPD group (61.4% compared to non-LBPD group, 51.9%, *p* = 0.879). “Sniffin Sticks” score demonstrated an OR of 0.715 (0.65–0.79; *p* < 0.001) for association with LBPD indicating lower “Sniffin Sticks” score predicted LBPD. RBD, depression, hallucination, and constipation symptoms were significantly higher in LBPD group than non-LBPD group, and all four symptoms showed the odds ratios of more than 2 for association with the LBPD group.

### 3.6. Receiver Operating Curve for “Sniffin Stick” Score and Threshold Based on Diagnosis at Follow-Up Visit

We identified “Sniffin Sticks” score of ≤8 as the cutoff value for “hyposmia” to best differentiate LBPD from non-LBPD group, with sensitivity of 77.2% and specificity of 60.9, and correctly classifying 69.8% of the cases (Receiver Operating Characteristic (ROC) area under curve of 0.747, *p* < 0.05).

### 3.7. Adjusted Odds Ratios in Association with LBPD Group

The hierarchical logistic regression model correctly classified 73.6% of the cases (Table 3). “Sniffin Sticks” score showed an adjusted OR of 0.72 (0.65–0.81; *p* < 0.001) in association with LBPD group, while depression and constipation had adjusted odds ratios of 2.15 (1.14–4.05; *p* = 0.017) and 3.53 (1.56–7.98; *p* = 0.002), respectively. Age of symptom onset did not serve as independent predictor when adjusted for other covariates. In the adjusted model, RBD and hallucinations showed odds ratios of 1.35 (0.62–2.94; *p* = 0.443) and 2.11 (0.81–5.46; *p* = 0.125), respectively, for association with the LBD group but did not reach statistical significance. The constructed regression model has a predictive power of 75.6% sensitivity and 71.0% specificity for LBPD, ROC area under curve of 0.799, and 95% confidence interval 0.75–0.85, *p* < 0.05.

## 4. Discussion

Our results demonstrated that diagnoses of Parkinsonian syndromes are challenging and far from accurate, especially by nonmovement disorder specialists. We found that, for instance, the referring doctor to the movement disorder clinics diagnosed or suspected fewer than half of cases ultimately proving to be PD. This finding is comparable to a previous study where only 32% of PD was diagnosed or suspected at first medical encounter [14]. Standard criteria focus on motor signs and levodopa responsiveness; however our data provides support for the utilization of history and examination for nonmotor symptoms and signs to improve earlier clinical detection of LBPD and detection of these features are likely to influence treatment.

Constipation has been less commonly studied nonmotor symptom in PD yet in our study it appeared to be the most useful discriminating symptom to separate LBPD from other Parkinsonism disorders in the early stages of the disease. This is despite the relatively high prevalence of constipation in healthy control populations and the potential for constipation to affect individuals with clinical diagnosis of MSA and PSP [15], although presumably constipation is not a common premotor symptom in atypical parkinsonian disorders [16].

Olfactory dysfunction in PD has long been recognized in literature [17]. The prevalence of hyposmia or anosmia in PD has been reported to range from 70% to 90% [17]. In PD, olfactory dysfunction may be identified regardless of whether the odour tests were targeted on detection, discrimination, or identification. DLB, which has shared clinical symptoms and pathological changes with PDD, is also associated with olfactory impairment [18]. Olfactory testing is useful to differentiate PD from atypical Parkinsonism such as MSA-P, Progressive Supranuclear Palsy (PSP), or Corticobasal Syndrome (CBS) as olfaction is usually preserved or mildly affected in these syndromes [19]. Olfactory function also has discriminatory value in vascular Parkinsonism in which smell preserved [20]. Olfactory testing can also be helpful to differentiate tremor-predominant PD from essential tremor ET [21] and dystonic tremor [22].

While olfactory testing can be simple and quick in the clinic, the clinical discrimination of PD from other Parkinsonian syndromes can be difficult. At present, a couple of olfactory testing kits, such as the University of Pennsylvania Smell Identification Test (UPSIT) [23] and the “Sniffin Sticks” test [24], have been made available but are not used widely beyond specialized centers for research purposes. While both tests have established validity in Parkinsonian syndromes and demonstrated usefulness in Australian populations [25], the validated four-minute twelve-item “Sniffin Sticks” test is a better office test for clinician as it incurs less cost and time to screen for olfactory dysfunction [6].

Our data showed that the mean “Sniffin Sticks” score was significantly lower in LBPD patients based on 12-item four-minute Sniffin Stick test, and we identified cutoff value of ≤8 as the most sensitive “hyposmia” level to separate LBPD from non-LBPD patients. Although the specificity of “Sniffin Sticks” test is only 60.9% when using the cutoff value of 8, it has sensitivity of 77.2%. In other words, about 8 out of 10 patients with LBPD could be correctly classified when their “Sniffin stick” score is 8 or less. It is however not specific and therefore should be used as an ancillary tool rather than diagnostic tool and should be used in the context of other clinical symptoms and presence of other nonmotor symptoms.

Moreover, we found that patients subsequently found to have DLB or PDD had significantly worse olfactory function at initial assessment compared to patients with PD at follow-up. As odour identification with four-minute “Sniffin Sticks” Test would require both intact olfaction and memory, the results may also provide a surrogate measure of cognitive function. This is consistent with current models of olfactory dysfunction in PD where neuroimaging studies in PD have indicated both structural and functional abnormality in olfactory system involving the amygdala and piriform cortex, areas which also play a role in memory functions [26].

There has been much interest in the concept of a premotor form of Parkinson's disease defined by a range of nonmotor symptomatologies, but the role of these nonmotor symptoms in PD diagnosis has received relatively little attention.

RBD is another recognized risk factor for subsequent development of neurodegenerative disease and could precede the illness for many years. Cohort studies have shown that risk of developing neurodegenerative disease could be as high as 65% in idiopathic RBD patients, especially in patients with concomitant anosmia [27]. The use of simple close-ended questions regarding symptom of RBD in our study is in accordance with the recent validation of single questionnaire for RBD by Postuma and colleagues, which demonstrated a high sensitivity of 94% [8]. RBD is a source of distress for patients and their carers and routine evaluation and treatment are important. Presence of RBD in cross-sectional cohorts is a useful clinical feature to differentiate the aforementioned diseases from other Parkinsonian disorders, especially PSP and secondary Parkinsonism. In our study, we found higher prevalence of RBD in LBPD and MSA patients, with adjusted odd ratio of 1.35 in LBPD group. But in this study RBD failed to stand as an independent predictor potentially because of its lower prevalence in this incident cohort and proposed neurobiological commonalities with other nonmotor symptoms such as poor olfaction [28].

Similarly, premotor depression has attracted a lot of attention. The prevalence of depression in PD has recently been estimated to be around 35% in PD patients but varies according to the methodological and diagnostic tools employed and the challenges of dissecting overlapping features of Parkinsonism with the somatic symptoms of depression [29]. Depression in PD has been linked to dysfunctional dopaminergic neurotransmitters pathways as well as synucleinopathy extending to other subcortical regions. A previous study has demonstrated the utility of a single questionnaire for depression after stroke with reported sensitivity of 86% and specificity of 78% [30] but the utility of a single question for depression employed in this routine context has not been validated. Our data suggest that clinicians diagnosing PD should be familiar with the symptoms of depression in PD patients and at least routinely employ a simple question about whether patient is depressed. Patients screening positive with a single questionnaire may then be evaluated with the appropriate depression rating scale as proper treatment can improve patient's quality of life and potentially reduce suicidal risk [29].

The aetiology of hallucinations is less straightforward and results from an interaction between the disease process and the pharmacological management. In our study, the association between LBPD and hallucinosis at disease onset did not survive adjustment but would serve as a useful discriminator in later disease stages.

We acknowledged the limitations of this study in which there is lack of pathological confirmation and potential for a misinterpretation on the respondent's behalf or a lack of specificity of the questioning.* Moreover, longer follow-up could have further improved classification of the final clinical diagnosis*. This limitation is inevitable due to the retrospective nature of our study design and the clinical setting in which the study was conducted. Our results are also limited to a single-center experience, although the relatively large number of “real world” patients makes it possible for our findings to be generalized to a routine movement disorder practice. Furthermore, these data indicate that the administration of the four-minute Sniffin Sticks test questioning for the presence of a range of nonmotor symptomatology is a helpful guide in the diagnostic algorithm and subsequent management. Patients should routinely be quizzed for a range of nonmotor symptoms associated with Parkinson's disease particularly depressive symptoms, hallucinations, constipation, and RBD.

## 5. Conclusion

Our study results showed that lower olfaction score based on four-minute “Sniffin Sticks” testing combined with nonmotor symptoms in particular depression and constipation is independent predictors for diagnosis of Lewy body Parkinsonian disorders (LBPD). Further studies with four-minute Sniffin Sticks olfaction test and other nonmotor symptoms as adjunct diagnostic tools are warranted.

## Figures and Tables

**Table 1:** Baseline characteristics.

Diagnosis *N*	PD (128)	PDD/DLB (30)	PTDs (57)	UP (23)	Others (53)	*p* value
Gender						
Male						
Number (%)	75 (58.6)	22 (73.3)	30 (52.6)	11 (47.8)	28 (52.8)	0.286^†^

Family history^*∗*^						
Number (%)	11 (8.6)	3 (10.0)	3 (5.3)	1 (4.3)	4 (7.5)	0.875^†^

Age of symptom onset (year, SD±)	62.1 ± 12.6	69.7 ± 10.5	60.4 ± 15.9	64.7 ± 14.9	55.6 ± 16.1	0.001^‡^

^*∗*^First- or second-degree relative with Parkinsonism.

^†^Chi square test, level of significance *p* < 0.05.

^‡^Kruskal Wallis test and *post hoc* test with Mann Whitney test with PD as reference group, level of significance, *p* < 0.0125.

PD: Parkinson's disease; PDD: Parkinson's disease dementia; DLB: dementia of Lewy bodies; PTDs: Parkinsonian and tremor disorders; UP: unclassified Parkinsonism; SD: standard deviation.

**Table 2:** Mean “Sniffin Sticks” score.

Diagnosis	Mean (SD±)	95% CI for mean	*U* ^*∗*^
PD	6.4 (3.0)	5.90–6.96	Reference group
PDD and DLB	4.2 (2.5)	3.30–5.17	1111^‡^
PTDs	8.6 (2.2)	8.00–9.19	2107^‡^
UP	8.2 (2.8)	7.00–9.43	971^†^
Others	8.9 (2.3)	8.20–9.49	1801^‡^

^*∗*^Kruskal Wallis test and *post hoc* test with Mann Whitney test with PD as reference group, level of significance *p* < 0.05.

^†^ *p* < 0.0125, ^‡^ *p* < 0.001.

**Table 3:** Unadjusted and adjusted odds ratios in association with LBPD group.

Nonmotor symptoms (*N*)	LBPD (PD/PDD/DLB) (158)	Non-LBPD (133)	Unadjusted odds ratios (95% CI; *p* value)	Adjusted odds ratios (95% CI; *p* value)
Age of symptom onset				
Mean year (SD±)	63.5 (12.6)	59.2 (16.1)	1.02 (1.00–1.04; 0.014)	1.00 (0.98–1.02; 0.879)

Gender				
Male				
Number (%)	97 (61.4)	69 (51.9)	1.48 (0.92–2.35; 0.103)	N/A

Family history^*∗*^				
Number (%)	14 (8.9)	8 (6.0)	1.52 (0.62–3.74; 0.363)	N/A

Sniffin Sticks score				
Mean (SD±)	6.1 (3.0)	8.7 (2.4)	0.72 (0.65–0.79; <0.001)	0.72 (0.65–0.81; <0.001)

RBD				
Number of patients (%)	43 (27.2)	16 (12.0)	2.73 (1.46–5.13; 0.002)	1.35 (0.62–2.94; 0.443)

Depression				
Number of patients (%)	55 (34.8)	26 (19.5)	2.20 (1.28–3.77; 0.004)	2.15 (1.14–4.05; 0.017)

Hallucination				
Number of patients (%)	28 (17.7)	11 (8.3)	2.39 (1.14–5.01; 0.018)	2.11 (0.81–5.46; 0.125)

Constipation				
Number of patients (%)	51 (32.3)	11 (8.3)	5.29 (2.62–10.66; <0.001)	3.53 (1.56–7.98; 0.002)

^*∗*^First- or second-degree relative with Parkinsonism.
